# Thumb CMCJ arthritis: a new technique of suspensionplasty (Mini tightrope)

**DOI:** 10.1186/1753-6561-9-S3-A51

**Published:** 2015-05-19

**Authors:** Caroline Leclercq

**Affiliations:** 1 Institut de la Main, 75016, Paris, France

## 

Osteoarthritis of the thumb CMC joint is the most frequent degenerative arthritis in the upper limb. It is a very common cause of pain, loss of strength and functional impairment of the hand, reported in approximately one in three postmenopausal females. Conservative treatment (analgesics, NSAIDs, splinting and corticosteroid injections) can be helpful, but fail to provide long term relief in many patients. Surgery is indicated in these cases.

Many different surgical techniques have been advocated, including simple (Wilson) or double (Goubeau) osteotomy, trapeziectomy alone or combined with suspensionplasty and / or tendon interposition (LRTI: the Burton- Pellegrini technique), implant arthroplasty, and arthrodesis. Although each of these treatments have their specific limitations, most of the results published in the literature have indicated a satisfactory pain relief, and varied degrees of decrease in pinch strength and in range of motion.

The suspensionplasty techniques aim at maintaining the trapezial height after trapeziectomy. They involve a ligamentoplasty utilizing part of the flexor carpi radialis or of the abductor pollicis longus. A new technique of suspensionplasty has been developed by Yao in 2010, which suspends the first metacarpal to the second by means of a strong suture material (fiberwire) introduced through both bones’ metaphyses, and tied with a suture button (Mini-TightRope).

This technique is less invasive and less time consuming than the previous ligamentoplasties, because there is no need to harvest a nearby tendon. Furthermore, active motion is initiated early on after surgery. A bulky dressing is applied for ten days, then gentle auto-exercises are performed by the patient him(her)self once a day. Rehabilitation is started after one month.

Results of this technique have been published at two years follow-up by Wells (25 cases), and by Yao (21 cases), showing excellent pain relief, pinch strength amounting to 80% of the contralateral side, and conservation of the range of motion. Radiological follow-up shows some degree of subsidence of the trapezial height over the first year, without any incidence on the clinical outcome.

Clinical results continue to improve over the first two years.

The rare specific complications reported with this technique are second metacarpal fractures, and subcutaneous prominence of the suture button on the second metacarpal shaft.

